# Influence of genetic co-factors on the population pharmacokinetic model for clopidogrel and its active thiol metabolite

**DOI:** 10.1007/s00228-017-2334-z

**Published:** 2017-09-15

**Authors:** Dorota Danielak, Marta Karaźniewicz-Łada, Anna Komosa, Paweł Burchardt, Maciej Lesiak, Łukasz Kruszyna, Agnieszka Graczyk-Szuster, Franciszek Główka

**Affiliations:** 1 Department of Physical Pharmacy and Pharmacokinetics, Poznan University of Medical Sciences, Święcickiego 6 St, 60-781 Poznań, Poland; 2 First Department of Cardiology, Poznan University of Medical Sciences, Poznań, Poland; 3 Department of Biology and Environmental Sciences, Poznan University of Medical Sciences, Poznań, Poland; 4 Department of Cardiology, J. Struś Hospital, Poznań, Poland; 5 Department of General and Vascular Surgery, Poznan University of Medical Sciences, Poznań, Poland

**Keywords:** Population pharmacokinetics, Pharmacogenetics, Prodrugs, Single nucleotide polymorphism, Clinical pharmacokinetics

## Abstract

**Purpose:**

A high interindividual variability is observed in the pharmacokinetics of clopidogrel, a widely used antiplatelet drug. In the present study, a joint parent-metabolite population pharmacokinetic model was developed to adequately describe observed concentrations of clopidogrel and its active thiol metabolite (H4).

**Methods:**

The study included 63 patients undergoing elective coronarography or percutaneous coronary intervention. The population pharmacokinetic model was developed in the NONMEM 7.3 software, and first-order conditional estimation method with interaction was applied. Also, the influence of covariates was evaluated (age, weight, body mass index (BMI), obesity defined as BMI ≥ 30 kg/m^2^, sex, diabetes mellitus, co-administration of PPI or statins, presence of CYP2C19*2, CYP2C19*17, CYP3A4*1G alleles, and *ABCB1 3435 TT* genotype).

**Results:**

It was found that the only significant covariate was the presence of CYP2C19*2 allele, which had an impact on lower conversion of clopidogrel to H4. As a result, predicted area under the time-concentration curve values was lower in carriers of this allele, with median 5.94 ng h/ml (interquartile range 3.92–12.51 [ng∙h/ml]) vs. 12.70 ng h/ml in non-carriers (interquartile range, 7.00–19.39 [ng∙h/ml]), respectively (*p* = 0.004).

**Conclusions:**

Developed model predicts that the only significant covariate influencing the observed concentrations and therefore the exposure to the active H4 metabolite is the presence of CYP2C19*2 allele.

**Electronic supplementary material:**

The online version of this article (10.1007/s00228-017-2334-z) contains supplementary material, which is available to authorized users.

## Introduction

Clopidogrel, a second-generation thienopyridine, is a widely used antiplatelet agent. According to the American College of Cardiology recommendations, the treatment with clopidogrel in combination with aspirin should be continued in patients with acute coronary syndromes for at least 12 months and for 6 months in patients with stable ischemic heart disease [1]. As showed in clinical trials, clopidogrel effectively lowers the occurrence of myocardial infarction, ischemic stroke, or vascular death in patients at risk of ischemic events [2]. However, the response to clopidogrel in the population is varied and it is estimated that in 16–50% of patients, a high on-treatment platelet reactivity is observed [3]. Hence, the inhibition of platelet aggregation is inadequate and the risk of major adverse cardiac events is higher.

Clopidogrel is a prodrug that requires biotransformation to the active metabolite. The majority of drug (85–90%) is metabolized to the carboxylic acid metabolite by carboxylesterases. Only a small portion of the drug undergoes a two-step activation process, which is mediated by cytochrome P-450 (CYP). In an in vitro experiment, Kazui et al. [4] successfully identified that in the first step, clopidogrel is transformed into 2-oxo-clopidogrel by CYP1A2, CYP2B6, and CYP2C19. Subsequently, the thiolactone ring is opened in a reaction catalyzed by CYP2B6, CYP2C9, CYP2C19, and CYP3A4, as shown in the Online Resource file. The product of this reaction, the thiol metabolite, irreversibly binds with P2Y_12_ receptor which is located on the platelet surface. As a result, the ADP-mediated platelet aggregation is inhibited [5]. Tuffal et al. [6] have found that two stereoisomers of the active thiol metabolite are present in vivo, labeled H3 and H4. However, only H4 isomer exerts the antiplatelet effect.

Numerous studies show that several genetic and non-genetic aspects might be influencing this phenomenon of resistance to clopidogrel. Most notably, the *CYP2C19* loss-of-function alleles (LoF) such as *2 and *3 are connected with poor response to clopidogrel, lower H4 metabolite concentrations, and higher risk of cardiovascular events [7–9]. This is particularly important in Asian populations, in which the *CYP2C19* LoF alleles are more prevalent [10]. Other genetic polymorphisms occurring in the gene sequences are also considered as potential factors of variable response to clopidogrel. As shown in the literature, *ABCB1 3435 TT* genotype might be associated with lower exposition to the parent drug [11]. Also, *CES-1* (carboxylesterase) *c.428 G > A* allele was shown to have an influence on higher concentrations of both clopidogrel, active metabolite, and better inhibition of platelet aggregation [12, 13]. Allelic variations in *CYP3A4* [14] and *P2RY12* [15, 16] genes were found to be associated with insufficient response to clopidogrel. Among the non-genetic factors, concomitant use of proton pump inhibitors (PPIs) is associated with the occurrence of adverse cardiovascular events [17]. Also, lower exposure to the active metabolite is observed when CYP2C19-metabolized PPIs, most notably omeprazole and esomeprazole, are co-administered with clopidogrel [18]. Other drugs, such as rosuvastatin, might have an influence on the higher on-treatment platelet reactivity [19].

The aims of this study were to develop a population pharmacokinetic model for clopidogrel and its H4 metabolite and to explore the influence of the most important covariates, including frequently occurring genetic polymorphisms.

## Experimental

### Patients and sampling protocol

The following study included 63 patients of Caucasian origin who received clopidogrel prior to elective coronarography or percutaneous coronary intervention (PCI) procedure. All subjects received oral 75-mg clopidogrel for at least 7 days prior to the procedure and sample collection. The exclusion criteria were as follows: acute myocardial infarction, treatment with a glycoprotein IIb/IIIa antagonist, coumarine derivatives or other antiplatelet drugs with exception of aspirin, platelet count < 100,000/μl, on-going malignancies and liver dysfunctions, impaired renal function (serum creatinine > 2 mg/dl). The study protocol was approved by the local Ethical Committee at Poznan University of Medical Sciences, and all procedures were in accordance with the 1964 Helsinki declaration and its later amendments or comparable ethical standards. Informed consent was obtained from all individual participants included in the study.

All sampling was performed at the day of the procedure. A full pharmacokinetic profile was obtained from 17 patients. The sampling times were as follows: 0.5, 1, 2, 3, and 4 h after clopidogrel administration. From the remaining 46 subjects, only two samples were collected, 0.5 and 2 h or 1 and 3 h after receiving the drug. A short time of the last sample collection was based upon the results of previous studies [20]. Very low concentrations of both clopidogrel and H4 were determined and they were mostly below the detection limit 4 h after administration of clopidogrel. Five-milliliter full blood aliquots were drawn into vacuum systems containing EDTA-K (Sarstedt, Germany). Immediately after collection, 25-μl aliquot of 500 mM bromo-3′-methoxyacetophenone solution was added to stabilize highly labile thiol metabolite, as described by Takahashi et al. [21]. According to Tuffal et al. [6], samples with poor signs of hemolysis might be insufficiently stabilized with the bromo-3′-methoxyacetophenone. Therefore, they were discarded from the analysis. Centrifuged plasma was stored at −25 °C until further analysis.

### Determination of clopidogrel and H4 metabolite

A validated HPLC-MS/MS method was applied for determination of clopidogrel and H4 [22]. The validation procedure was conducted according to the guideline issued by the European Medicines Agency [23]. The method was linear within 0.25–5 ng/ml for clopidogrel and 0.25–50 ng/ml for derivatized H4, respectively. Within- and between-day precision, expressed as a relative standard deviation, was below 19.9% for both analytes, while the relative error of the assay was below 16%.

### Determination of genetic polymorphisms

From the collected samples, genomic DNA was extracted with a Quick Blood Purification Kit (EurX, Poland), according to the procedure supplied by the manufacturer. Four SNPs were evaluated: CYP2C19*2 (rs4244285), CYP2C19*17 (rs12248560), CYP3A4*1G (rs2242480), and *ABCB1 3435C > T* (rs1045642). CYP2C19*2, CYP3A4*1G, and *ABCB1 3435C > T* were determined by means of polymerase chain reaction with restriction fragment length polymorphism analysis (PCR-RFLP). CYP2C19*17 was determined with an allele-specific PCR. The conditions of the reactions were described in details elsewhere [9, 11, 24, 25].

### Population pharmacokinetic analysis

#### Software and methods

The joint population pharmacokinetic model for clopidogrel and H4 was developed by means of NONMEM software package (version 7.3.0, ICON Development Solutions, Hanover, MD, USA). ADVAN5 or ADVAN6 subroutines were used in linear or non-linear models, respectively. First-order conditional estimation method with interaction (FOCE-I) was applied. Diagnostic plots were generated with the R program (version 3.1.2, Foundation for Statistical Computing, Vienna, Austria) and Xpose (version 4.5.3, Uppsala University, Sweden). Scripts implemented in the Perl Speaks NONMEM (PsN, version 4.4.0) [8] were used for visual predictive check (VPC), covariate searching, and model validation. All modeling and simulation were run through a Pirana graphical user interface (version 2.9.2) [9]. An improvement in the model fit was evaluated with the likelihood ratio test. A difference in the objective function value (OFV) of 3.84 (*p* < 0.05) between nested models was considered significant, when one variable was added into the model. Also, a visual examination of the diagnostic plots was used to determine the model fit. For each tested model, the following plots were inspected: individual- and population-predicted concentrations versus observed concentrations, individual- and population-predicted concentrations versus time, weighted (WRES) and conditional-weighted residuals (CWRES) versus predicted concentrations, WRES and CWRES versus time, distribution of CWRES. Data points with │CWRES│ > 4 were considered as outliers. The outlying points were carefully examined and were only removed from the dataset when they were considered as improbable from pharmacokinetic point of view. The interindividual variability (IIV) elements for the pharmacokinetic parameters were applied exponentially:

$$ {\theta}_{ij}={\theta}_j\times {e}^{\eta_{ij}} $$

where *θ*_*ij*_ is a value of j-th pharmacokinetic parameter for i-th individual, *θ*_*j*_ is the population parameter estimate, and *η*_*ij*_ is a random variable characterizing IIV, which is normally distributed with mean zero and variance of ω^2^. Correlations between IIV elements were inspected and included in the structural model.

Additive, proportional, and combined error models for describing the residual variability (RV) were tested. The following equation was applied:

$$ {C}_{\mathrm{obs}}={C}_{\mathrm{pred}}\times \left(1+{\varepsilon}_1\right)+{\upvarepsilon}_2 $$

where *C*_obs_ and *C*_pred_ are observed and predicted concentrations, *ε*_1_ is a variable associated with proportional RV (removed from the equation while testing the additional error model), and *ε*_2_ defines additive portion of RV (removed from the equation while testing the proportional error model).

For the concentrations below the quantitation limit, an M3 method was applied [26, 27].

#### PK model development

In the first step of the model development, a structural model was determined. Initially, the base model for clopidogrel was established, which included an absorption compartment and a central compartment. Subsequently, further compartments were added to characterize the metabolite pharmacokinetics. Noteworthy, the determined H4 concentrations were adjusted to the mass equivalent of the parent compound. To adequately present the complex absorption and metabolite-formation process, several issues were tested: first-pass effect, absorption lag time, zero- and first-order linear kinetics, non-linear kinetics of metabolite formation, inclusion of transition compartments. It was assumed that the formation of the H4 metabolite was irreversible.

#### Covariate analysis

Potential covariates were evaluated after visual inspection of their possible relationships with PK parameters included in the model. Then, a covariate model was created in a stepwise forward-inclusion backward-elimination manner with a PsN *scm* script. A 3.84 or greater drop in the OFV (*p* ≤ 0.05) was chosen as a threshold for covariate inclusion. After all significant covariates were included, a full model was obtained. In the next step, the covariates were sequentially eliminated. A covariate was retained in a final model, if the OFV increased by more than 6.63 (*p* ≤ 0.01). Also, clinical significance of the covariates was considered. The following covariates were inspected: age, weight, body mass index (BMI), obesity defined as BMI ≥ 30 kg/m^2^, sex, diabetes mellitus, co-administration of PPI or statins, presence of CYP2C19*2, CYP2C19*17, CYP3A4*1G alleles, and *ABCB1 3435 TT* genotype. For continuous covariates, a linear, piece-wise, exponential, and power parameter-covariate relations were tested. Categorical covariates were linearly included.

#### Simulations and model diagnostics

VPC diagnostics was performed for 1000 simulated observations, and 90% prediction intervals (PI) were constructed from simulated concentration-time profiles and compared with observed data. Additionally, 1000 datasets were bootstrapped, and as a result, median value of each PK parameter, as well as 5th and 95th confidence intervals (CI) was obtained. Prior to bootstrapping, the dataset was stratified upon the number of samples obtained from each subject (17 full profiles vs 46 sparingly sampled) and upon the categorical covariates retained in the final model.

### Estimation of exposure to clopidogrel and H4

Model-based estimation of the area under the time-concentration curve (AUC) for both the clopidogrel and H4 was based on the obtained empirical Bayes estimates of apparent clearances as follows:

$$ {\displaystyle \begin{array}{c}\hfill {AUC}_{CL P}=\frac{F\times D}{CL_{CL P}}\hfill \\ {}\hfill {AUC}_{H4}=\frac{FM\times F\times D}{CL_{H4}}\hfill \end{array}} $$where *AUC*_*CLP*_ and *AUC*_*H*4_ are AUC of clopidogrel and the active metabolite, respectively, *F* is bioavailability, *D* is the dose of clopidogrel (75 mg), *FM* is a fraction of clopidogrel converted to H4, and *CL*_*CLP*_ and *CL*_*H*4_ stand for the clearances of the clopidogrel and H4, respectively.

The AUC comparison in the presence of influential covariates was performed with a non-parametric *U* Mann-Whitney’s test in the Statistica 12 software (Statsoft, Inc. 2014).

## Results

### Patients’ characteristics and genotype frequencies

The characteristics of patients, for whom quantifiable concentrations of clopidogrel and H4 were available, are presented in Table 1. Since pantoprazole was almost exclusively administered as a PPI of choice, the discrimination between the types of PPIs was not considered in this analysis. The final dataset consisted of 155 clopidogrel concentrations and 158 H4 concentrations. A total of 17 samples were below quantitation level (10 for clopidogrel and 7 for H4). Three hours after drug administration, 6.5% of clopidogrel and 6.4% of H4 concentrations were below quantitation level, while 4 h after drug administration below quantitation threshold were 35.7 and 28.6% of clopidogrel and H4 concentrations, respectively. For 1 woman and 7 men, the data on bodyweight and BMI was missing. For further covariate analysis, the missing data were imputed with mean values. For female patients, the means were 73.5 kg for weight and 29.88 kg/m^2^ for BMI, respectively, while 82.3 kg weight and 26.90 kg/m^2^ BMI were imputed for male patients.

**Table 1:** Patients’ demographics and genotype distribution (*n* = 63). Age, weight, and BMI are presented as mean with standard deviation. Categorical data are presented as quantities with frequencies

Parameter	Value
Age [years]	65.4 ± 10.5
Weight [kg] (*n* = 55)	79.1 ± 14.0
BMI [kg/m^2^] (*n* = 55)	28.40 ± 4.89
BMI ≥ 30	37 (67.3%)
Sex (male/female)	42 (66.7%)/21 (33.3%)
DM	22 (34.9%)
Statins	60 (95.2%)
PPI	46 (73.0%)
Pantoprazole	43 (93.5%)^a^
Omeprazole	2 (4.3%)^a^
Esomeprazole	1 (2.2%)^a^
Allele	Number of carriers
CYP2C19*2	20 (31.7%)
CYP2C19*17	34 (54.0%)
CYP3A4*1G	10 (15.8%)
*ABCB1 3435TT*	22 (34.9%)
Phenotype	Number of patients
UM (*1/*17 and *17/*17)	25 (39.7%)
EM (*1/*1)	18 (28.6%)
IM (*1/*2)	20 (31.7%)

*BMI* body mass index, *DM* diabetes mellitus, *PPI* proton pump inhibitors, *UM* ultrarapid metabolizers, *EM* extensive metabolizers, *IM* intermediate metabolizers

^a^ The ﻿fr﻿equency ﻿of patients treated ﻿with *PPI*

### PK model

**Fig. 1:**
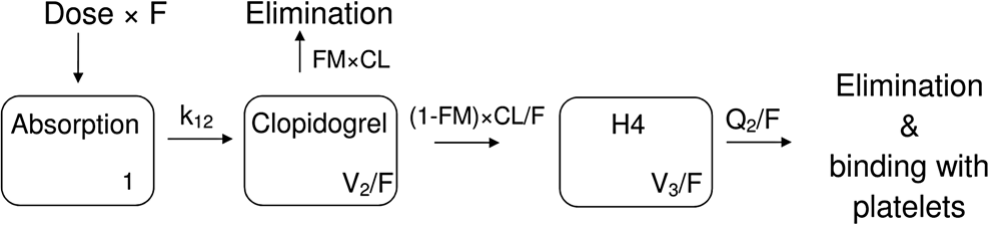
Final structural model for clopidogrel and H4

The final base structural model for pharmacokinetics of clopidogrel and H4 is presented in Fig. 1. Previous studies showed that clopidogrel undergoes extensive first-pass metabolism to the inactive carboxylic acid [28]. Inclusion of a first-pass effect rate constant from the depot compartment to H4 metabolite compartment did not improve the model (Supplementary Material Table 1). Since it is estimated that approximately 85% of the absorbed dose is inactivated in this process, the fraction of the drug converted to the active metabolite (FM) was constrained to be no larger than 20%. The relative bioavailability (F) of clopidogrel, described as a relative proportion of the drug absorbed after oral administration, was assumed a typical value of unity. The derived population base model consisted of log-normal distribution of IIV for k_12_, V_2_/F, CL/F, and FM. The model included covariance between k_12_ and V_2_/F. For clopidogrel and H4, a proportional error model, with separate terms for both entities, was applied. It was found that first-order kinetics best described transition of the drug between the compartments. Also, inclusion of additional peripheral or transition compartments did not improve the model fit.

The performed covariate analysis identified the presence of CYP2C19*2 as the most significant covariate that influenced the pharmacokinetics of the studied drug. In the first step of the covariate modeling, the following parameter-covariate relationships were found to be significant: k_12_–sex; CL/F–*ABCB1 TT*; FM–CYP2C19*2, CYP2C19*17; V_2_/F–sex. However, after including CYP2C19*2 as a cofactor for FM, the influence of the other covariates was negligible. Incorporation of this parameter improved the overall model fit and lowered the OFV, and therefore, it was retained in the final model. Final PK estimates derived from the final PK model are presented in Table 2. Detailed results of the covariate selection process are presented in the Online Resource file.

**Table 2:** Estimates of CLP and H4 pharmacokinetic parameters obtained from the final pharmacokinetic model with residual standard errors (RSE) and bootstrap results with 95% confidence intervals (CI)

Parameter	Final model	Bootstrap results	
	Estimate (RSE, %)	Mean	95% CI
k_12_ [1/h]	0.592 (3.53)	0.592	0.556–0.626
V_2_/F [L]	7660 (4.3)	7700	7310–8240
CL/F [L/h]	14,500 (3.9)	14,400	13,250–14,970
FM	0.045 (3.6)	0.045	0.044–0.048
V_3_/F [L]	4.89 (4.5)	4.90	4.65–5.15
Q_2_/F [L/h]	252 (4.2)	251	232–264
IIV^a^ for k_12_	25.3 (7.3)	24.9	23.7–26.9
IIV^a^ for V_2_/F	63.7 (19.6)	63.7	51.8–76.0
IIV^a^ for CL/F	49.8 (8.3)	47.1	45.9–53.5
IIV^a^ for FM	71.4 (7.9)	71.3	65.0–76.4
Effect of CYP2C19*2			
on FM (COV)^b^	−0.45 (8.4)	−0.45	−0.48–−0.41
Covariance between			
k_12_ and V_2_/F	−0.087 (49.3)	−0.076	−0.116–0.011
Proportional residual error for CLP	−0.45 (4.5)	−0.45	−0.49–−0.42
Proportional residual error for H4	−0.66 (5.4)	−0.65	−0.68–−0.58

^a^%CV = (SQRT(EXP(OMEGA(N))-1))*100%

^b^FM = TVFM*(1 + CYP2C19*COV)

**Fig. 2:**
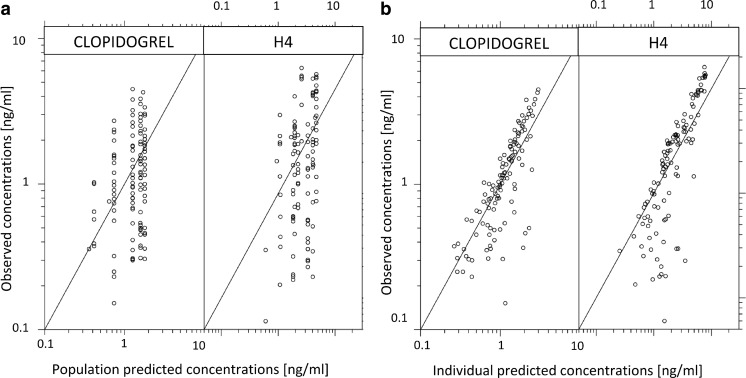
Diagnostic goodness-of-fit plots for clopidogrel and H4. **a** Observed concentrations versus population-predicted concentrations. **b** Observed concentrations versus individual-predicted concentrations

Goodness-of-fit plots presenting the correlations between individual- and population-predicted and observed concentrations of clopidogrel and H4 are presented in Fig. 2. The data points in population- and individual-predicted concentrations versus observed concentrations plots are widely spread around the identity line, reflecting a large unexplained RV. Additional diagnostic plots are presented in the Online Resource file.

**Fig. 3:**
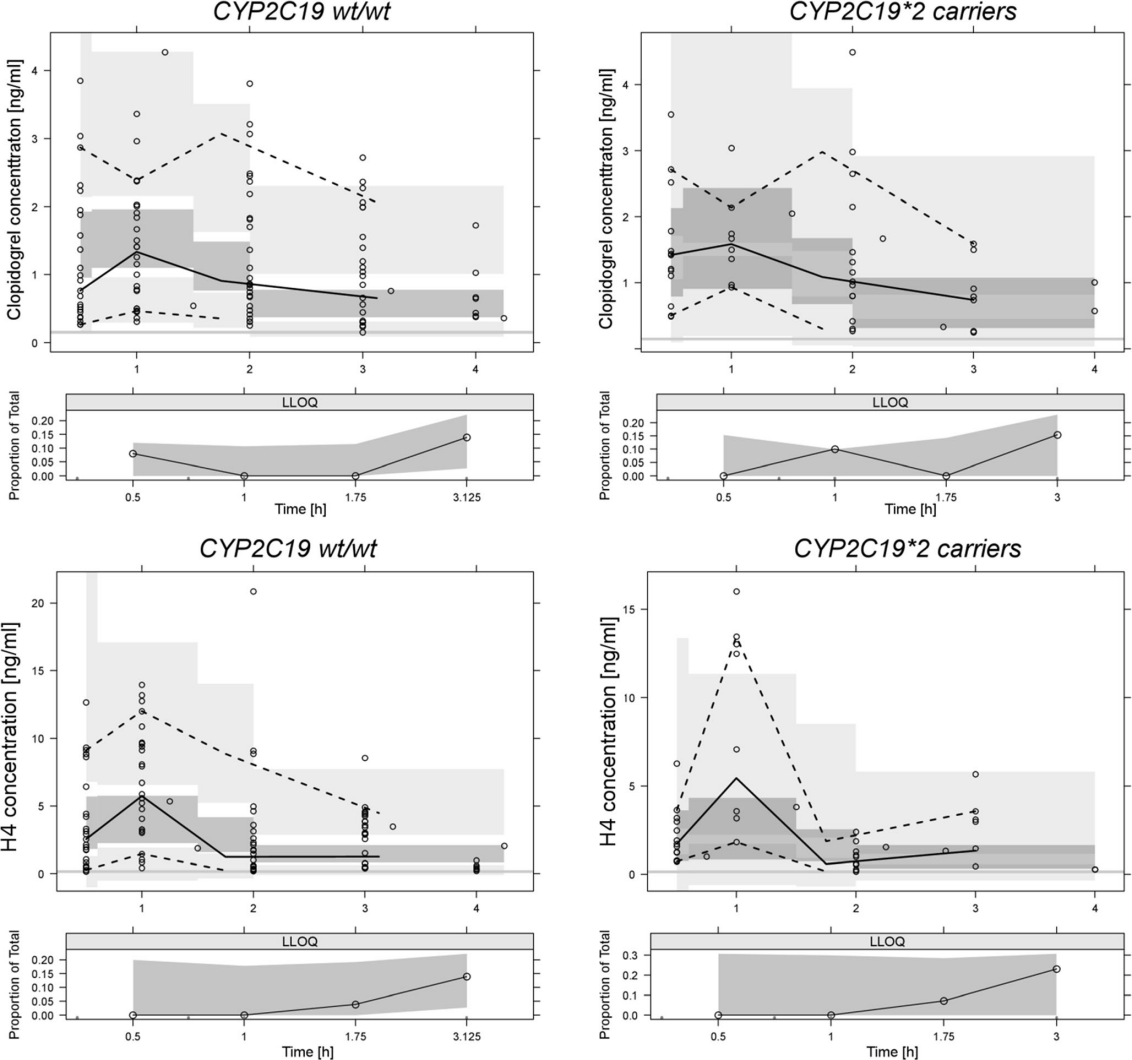
VPC plots for clopidogrel and H4 stratified upon the presence of CYP2C19*2 allele. Observed concentrations are presented as circles, bold line as median of the observed concentrations, and dashed lines as 95% confidence intervals of the observed concentrations. Dark gray areas are 50% prediction intervals of the simulated data and light gray areas are 90% prediction intervals of the simulated data

VPC, which graphically illustrates the model adequacy, is presented in Fig. 3. H4 concentrations were noticeably lower in carriers of CYP2C19*2 allele, while the concentrations of clopidogrel were similar in carriers of both wild-type (*wt*) and polymorphic *CYP2C19* alleles. Ninety percent interpercentile range for predicted data were utilized, corresponding to 5th and 95th percentiles of observed data. In small datasets, this approach is preferable to a more widely used 95% prediction intervals [29]. 4.8% of the observed clopidogrel concentrations and 4.2% of observed H4 concentrations fell outside the 90% prediction intervals. The resemblance of observed and simulated profiles is visible for both clopidogrel and H4. The simulated and observed medians are close to each other in almost all bins. The VPC for concentrations below quantitation limit shows that the concentrations of clopidogrel and H4 might be undetectable in as much as 20% of samples collected 3 h or later after the drug administration.

### Comparison of exposure to clopidogrel and H4

**Fig. 4:**
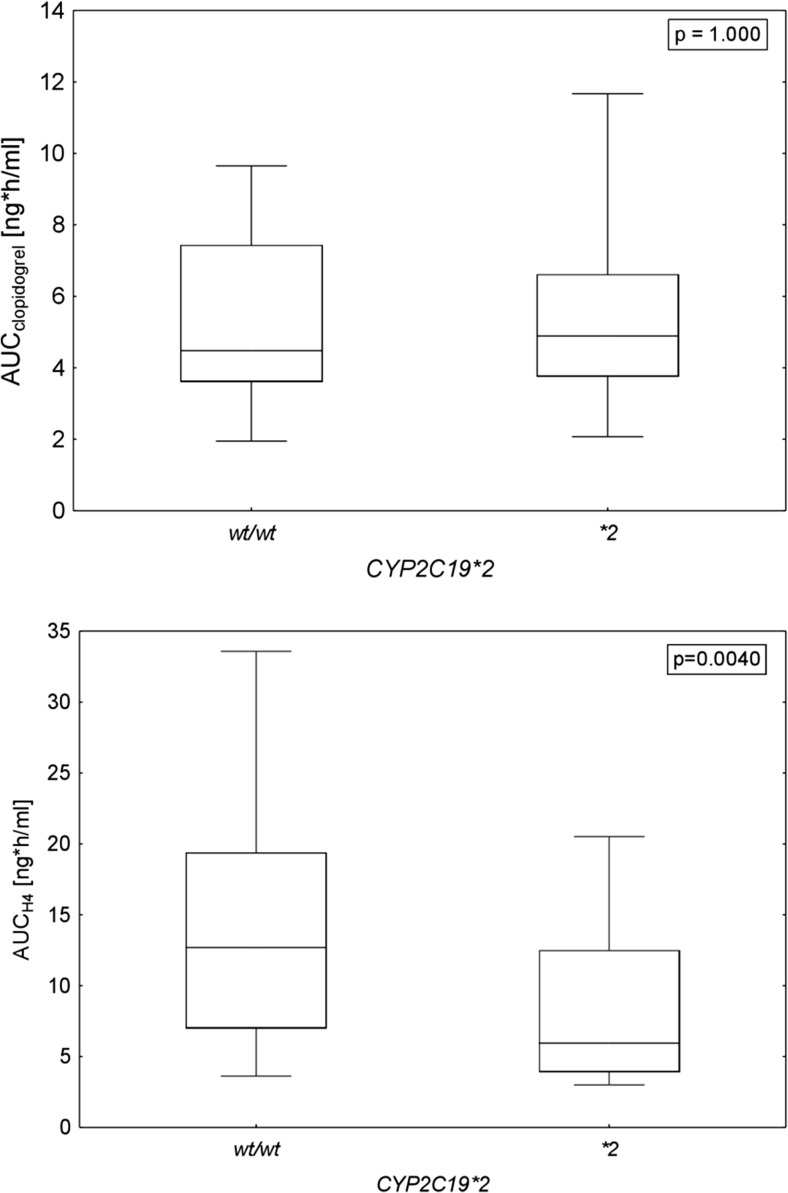
Comparison of the area-under-time-concentration curves (AUC) of clopidogrel and H4 in wild-type homozygotes (*wt/wt*) and CYP2C19*2 carriers. Data are presented as medians with boxed interquartile range and minimum-maximum as whiskers

The results of Mann-Whitney’s *U* test showed that the AUC of clopidogrel was not significantly different between carriers of CYP2C19*2 allele and wild-type homozygotes (Fig. 4a). In contrast, as shown in Fig. 4b, the presence of CYP2C19*2 loss-of-function allele was associated with noticeably lower AUC of the active H4 metabolite (13.58 ± 7.10 vs. 8.59 ± 6.48 ng h/ml, *p* = 0.004).

## Discussion

The pharmacokinetics of clopidogrel might be influenced by many factors, both genetic and non-genetic. Several population models have been established for describing pharmacokinetics and pharmacodynamics of clopidogrel. However, some of the previously published models are based upon measurements of the inactive carboxylic acid metabolite [30, 31]. Ernest et al. [32] have established a model that enabled prediction of the thiol metabolite concentrations. Yet it was not specified whether the reported concentrations reflect H3 and H4 isomers or only the H4 isomer which has the antiplatelet activity. Moreover, no data on the important genetic covariates and concentrations of the parent drug were included in that analysis. Recently, more elaborated physiologically based models were developed, that allowed the prediction of clopidogrel pharmacokinetics, yet they are very complicated and require numerous assumptions [33, 34]. Therefore, the present study was aimed at creating a joint parent-metabolite population pharmacokinetic model to explore the potential co-factors and to define the most influential ones.

In the present study, both clopidogrel and the H4 metabolite kinetics could be described with one-compartment model. The observed IIV for the parameters was very high (25.3–71.4%). It was also found that the linear elimination of both entities was sufficient to describe the observed concentrations.

The typical population value of FM was estimated to be less than 5%. It is in contrast with the previously mentioned model [32], which followed the assumption that all of the absorbed dose was metabolized to the active metabolite, due to lack of the data on the concentrations of the parent entity. The majority of clopidogrel undergoes an extensive first-pass metabolism to the inactive carboxylic acid (Online Resource file). However, the extent of this reaction, catalyzed by the CES-1 enzyme, could not be established in this study. Also, in the present study, linear model more adequately described the conversion to the H4 then non-linear one. In the recently published physiologically based models, this reaction was following Michaelis-Menten non-linear kinetics [33, 34]. The observation from the present study might be explained by the fact that the study group involved patients who were being administered 75 mg clopidogrel daily, and the observed maximum total plasma concentrations of clopidogrel did not exceed 5 ng/ml (0.015 μM). Therefore, they are much lower than the Michalis-Menten constants (K_m_) for the enzymatic conversion of the clopidogrel to H4 by each of the involved CYP450 isoenzymes, as established by other authors [4, 34]. However, since a full pharmacokinetic profile could be obtained from 17 patients only and both the clopidogrel and H4 are rapidly eliminated, the data might not be sufficient to adequately support a more complex model.

Since many factors, both genetic and non-genetic factors, might influence pharmacokinetics of clopidogrel, the impact of following potential covariates was evaluated: age, weight, sex, diabetes mellitus, co-administration of PPI or statins, the presence of CYP2C19*2, CYP2C19*17, CYP3A4*1G alleles, and *ABCB1 3435 TT* genotype. The most significant of the examined covariates was the presence of CYP2C19*2 allele. It was found that the FM was lower in the *2 carriers. This finding is consistent with numerous studies, which reported a strong association of *2 with lower exposure to the H4 metabolite [9, 35–37]. As a consequence, predicted AUC of the H4 metabolite was 36.7% lower in these patients, while the AUC of the parent drug remained unchanged (Fig. 4). This is in accordance with the predictions from the physiological-based models, in which intermediate (*1/*2) and poor metabolizers (*2/*2) have lower concentrations of the active metabolite [33, 34]. As indicated by other researchers, the exposure to the H4 metabolite can be over 30% lower in carriers of at least one *CYP2C19* LoF allele [36, 38]. In the present study, none of the patients could be classified as poor metabolizers; therefore, patients’ classification by phenotype was not performed. The other studied *CYP2C19* allele, labeled *17, is associated with an increased activity of CYP2C19 isoenzyme. In several studies, the *17 allele was related to lower on-treatment platelet reactivity and even with a “protective” effect [39, 40]. In the proposed model, the presence of this allele was a significant covariate for both FM and V_2_/F. However, after including the CYP2C19*2 as a most significant cofactor, the *17 was not influential. This might confirmed results from other studies which did not find *17 allele influencing H4 pharmacokinetics [41, 42].

*ABCB1 3435 TT* genotype is associated with an increased risk of major adverse cardiovascular events, as compared with *CC* and *CT* genotypes [43]. In several pharmacokinetic studies, it was shown that in the *TT* homozygotes, the exposure to the unchanged clopidogrel is significantly lower [11, 44, 45]. In the present study, step-wise analysis showed that *TT* genotype was a significant covariate for CL/F, and in its presence, the apparent clearance of clopidogrel was higher. However, after inclusion of CYP2C19*2 genotype, further addition of *ABCB1 TT* did not improve the model fit. Therefore, it might be concluded that this particular P-gp polymorphism is not an independent factor influencing clopidogrel pharmacokinetics. Similarly, Carlquist et al. [46] found that *ABCB1 3435C > T* polymorphism might be associated with the occurrence of cardiovascular events only when CYP2C19*2 is also considered.

Other covariates were not found to be of significant influence. Although some authors mention that covariates such as BMI [47] or obesity [48] might influence pharmacokinetics of clopidogrel or H4 metabolite, it was not confirmed in the present analysis.

In conclusion, the developed model predicts that the only significant covariate influencing the observed concentrations and therefore the exposure to the active H4 metabolite is the presence of CYP2C19*2 allele.

## Electronic supplementary material

ESM 1 (DOCX 228 kb).
