# Supplementary material for: Influence of genetic co-factors on the population pharmacokinetic model for clopidogrel and its active thiol metabolite
Source: Eur J Clin Pharmacol. 2017 Sep 15;73(12):1623–32. doi: 10.1007/s00228-017-2334-z (PMC5684285; doi:10.1007/s00228-017-2334-z)
Supplement: Supplementary file 1 — (DOCX 228 kb). [file 228_2017_2334_MOESM1_ESM.docx]

European Journal of Clinical Pharmacology

Electronic Supplementary Material

INFLUENCE OF GENETIC CO-FACTORS ON THE POPULATION PHARMACOKINETIC MODEL FOR CLOPIDOGREL AND ITS ACTIVE THIOL METABOLITE

Dorota Danielak^1^, Marta Karaźniewicz-Łada, Anna Komosa, Paweł Burchardt, Maciej Lesiak, Łukasz Kruszyna, Agnieszka Graczyk-Szuster, Franciszek Główka

^1^ Department of Physical Pharmacy and Pharmacokinetics, Poznan University of Medical Sciences, Poznań, Poland, e-mail: [danielak@ump.edu.pl](mailto:danielak@ump.edu.pl)

Supplementary Figure 1. Clopidogrel metabolic pathway. The CYP-450 enzymes involved in the metabolism are presented according to Kazui et al. (Drug Metab. Dispos. 2010;38:92–9.). CES-1 - carboxylesterase 1.


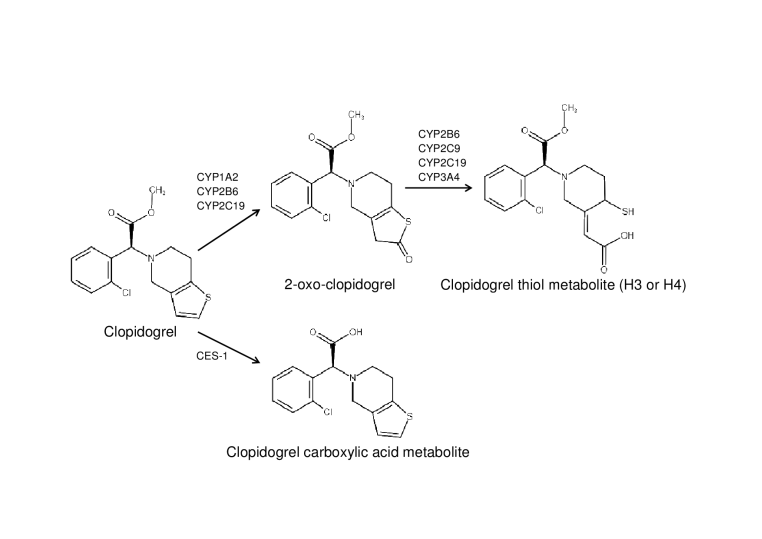


Supplementary Figure 2: A, Conditional weighted residuals (CWRES) versus time. B, CWRES versus predicted concentrations. The solid lines represent the line of identity in A and B, and the line y=0 in C and D.


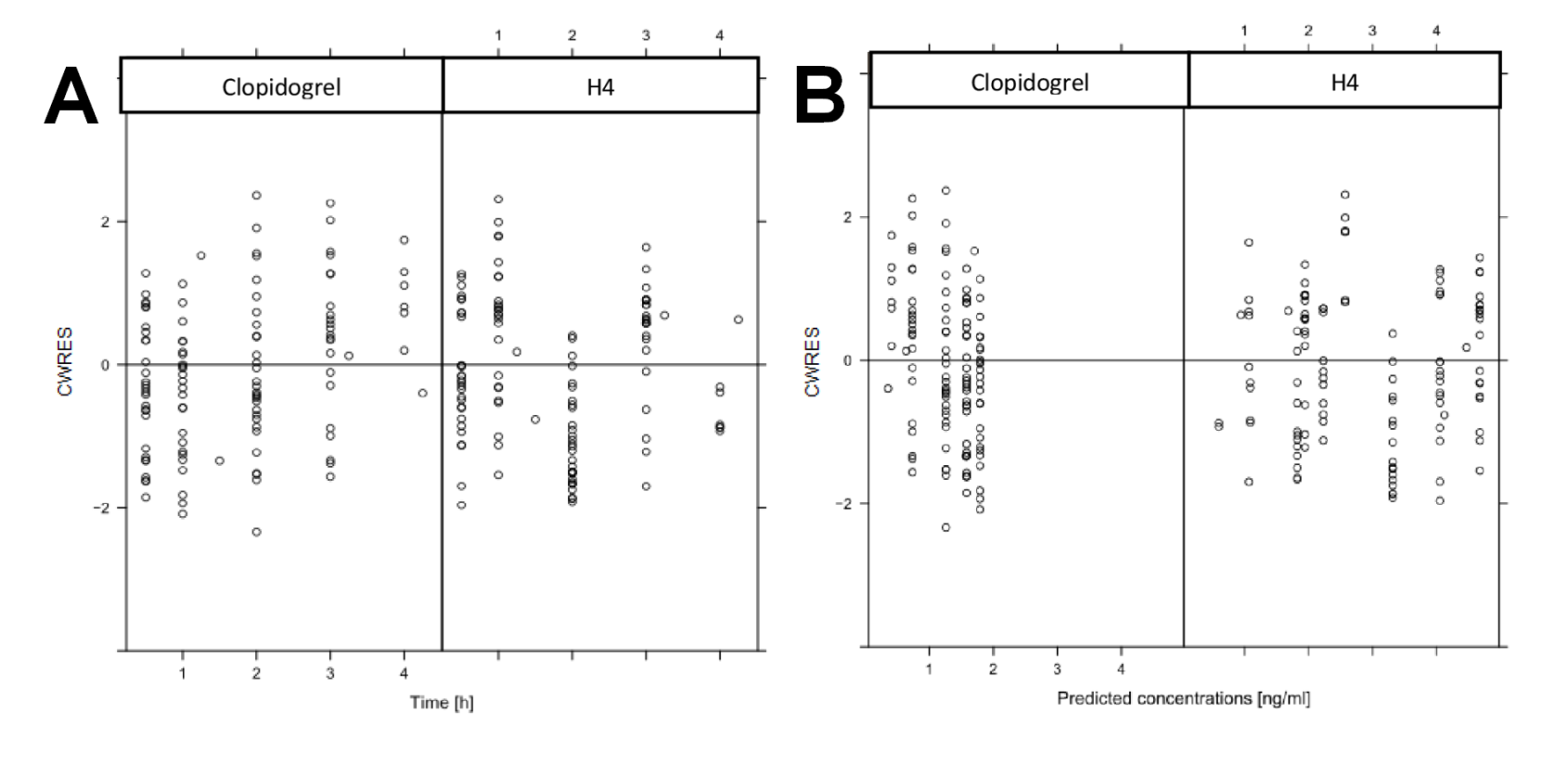


Supplementary Table 1: Optimization of model structure prior to covariate analysis.

| Model |  | OFV | dOFV | Included? |
| --- | --- | --- | --- | --- |
| Base model | K_a_ = θ_1_ × e^η^_1_  CL = θ_2_ × e^η^_2_  V2/F = θ_3_ × e^η^_3_  FM = θ_4_ × e^η^_4_  V3/F = θ_5_ × e^η^_5_  Q/F = θ_6_ | 506.99 | - | - |
| Addition of lag time | ALAG1 = θ_7_ | 510.930 | 3.94 | No |
| Introduction of first pass-metabolism | K13 = θ_7_ | 516.045 | 9.055 | No |
| Fixed compartment volumes | V2 = V3 | 824.499 | 318.813 | No |
| Non-linear metabolism of clopidogrel to H4 | KM = θ_7_  VMAX = θ_8_ | 658.065 | 151.672 | No |

OFV – objective function value, dOFV – difference in the objective function value

Supplementary Table 2. Step 1 of a forward-inclusion backward-elimination covariate selection.

| Pharmacokinetic parameter | Covariate | Drop in OFV | p | Significant? |
| --- | --- | --- | --- | --- |
| CL/F | AGE | 0.18742 | 0.6651 |  |
|  | WEIGHT | 0.95058 | 0.3296 |  |
|  | BMI | 0.44010 | 0.5071 |  |
|  | OBESITY | 0.36178 | 0.5475 |  |
|  | SEX | 0.80401 | 0.3699 |  |
|  | DIABETES MELLITUS | 0.25300 | 0.6150 |  |
|  | PPI | 0.15739 | 0.6916 |  |
|  | STATINS | 0.21398 | 0.6437 |  |
|  | CYP2C19*2 | 0.58309 | 0.4451 |  |
|  | CYP2C19*17 | 0.52110 | 0.4704 |  |
|  | ABCB1 3435 TT | 4.53027 | 0.0333 | YES |
|  | CYP3A4*1G | 2.79143 | 0.0948 |  |
| FM | AGE | 0.74827 | 0.3870 |  |
|  | WEIGHT | 0.02316 | 0.8790 |  |
|  | BMI | 0.51448 | 0.4732 |  |
|  | OBESITY | 0.85586 | 0.3549 |  |
|  | SEX | 1.03016 | 0.3101 |  |
|  | DIABETES MELLITUS | 0.00597 | 0.9384 |  |
|  | PPI | 0.20144 | 0.6536 |  |
|  | STATINS | 0.03331 | 0.8552 |  |
|  | CYP2C19*2 | 14.49229 | 0.0001 | YES |
|  | CYP2C19*17 | 5.64373 | 0.0175 | YES |
|  | ABCB1 3435 TT | 1.64051 | 0.2002 |  |
|  | CYP3A4*1G | 2.1522 | 0.1424 |  |
| K_12_ | AGE | 0.74827 | 0.3870 |  |
|  | WEIGHT | 1.2655 | 0.2606 |  |
|  | BMI | 0.06895 | 0.7929 |  |
|  | OBESITY | 0.23077 | 0.6310 |  |
|  | SEX | 5.11594 | 0.0237 | YES |
|  | DIABETES MELLITUS | 3.46269 | 0.0628 |  |
|  | PPI | 0.28910 | 0.5908 |  |
| V2/F | AGE | 1.72706 | 0.1888 |  |
|  | WEIGHT | 2.60213 | 0.1067 |  |
|  | BMI | 0.23794 | 0.6257 |  |
|  | OBESITY | 0.34052 | 0.5595 |  |
|  | SEX | 5.45637 | 0.0194 | YES |
|  | DIABETES MELLITUS | 0.98102 | 0.3219 |  |
| V3/F | AGE | 0.25529 | 0.6134 |  |
|  | WEIGHT | 0.00116 | 0.9729 |  |
|  | BMI | 2.01532 | 0.1557 |  |
|  | OBESITY | 0.07283 | 0.7873 |  |
|  | SEX | 0.11846 | 0.7307 |  |
|  | DIABETES MELLITUS | 0.18695 | 0.6655 |  |

Supplementary Table 3. Step 2 of a forward-inclusion backward-elimination covariate selection, after including CYP2C19*2 as a covariate on FM

| Pharmacokinetic parameter | Covariate | Drop in OFV | p | Significant? |
| --- | --- | --- | --- | --- |
| CL/F | AGE | -1.98190 | - |  |
|  | WEIGHT | -1.07394 | - |  |
|  | BMI |  |  |  |
|  | OBESITY |  |  |  |
|  | SEX | -0.33616 | - |  |
|  | DIABETES MELLITUS | -0.06814 | - |  |
|  | PPI |  |  |  |
|  | STATINS |  |  |  |
|  | CYP2C19*2 | -0.27884 | - |  |
|  | CYP2C19*17 | -1.04557 | - |  |
|  | ABCB1 3435 TT | -2.07771 | - |  |
|  | CYP3A4*1G | 1.77473 | 0.1828 |  |
| FM | AGE | -1.68202 | - |  |
|  | WEIGHT | -1.75417 | - |  |
|  | BMI |  |  |  |
|  | OBESITY |  |  |  |
|  | SEX | -2.17480 |  |  |
|  | DIABETES MELLITUS | 0.35216 | 0.5529 |  |
|  | PPI |  |  |  |
|  | STATINS |  |  |  |
|  | CYP2C19*17 | 0.41426 | 0.5198 |  |
|  | ABCB1 3435 TT | 2.47309 | 0.1158 |  |
|  | CYP3A4*1G | -0.4354 | - |  |
| K_12_ | AGE | -2.03425 | - |  |
|  | WEIGHT | -0.21861 | - |  |
|  | BMI |  |  |  |
|  | OBESITY |  |  |  |
|  | SEX | -1.42025 | - |  |
|  | DIABETES MELLITUS | 1.32519 | 0.2497 |  |
|  | PPI |  |  |  |
| V2/F | AGE | -2.19929 | - |  |
|  | WEIGHT | -1.59112 | - |  |
|  | BMI |  |  |  |
|  | OBESITY |  |  |  |
|  | SEX | -1.36292 | - |  |
|  | DIABETES MELLITUS | 0.65973 | 0.4166 |  |
| V3/F | AGE | 0.64199 | 0.4230 |  |
|  | WEIGHT | 0.67633 | 0.4108 |  |
|  | BMI |  |  |  |
|  | OBESITY |  |  |  |
|  | SEX | -0.25325 | - |  |
|  | DIABETES MELLITUS | 0.17395 | 0.6766 |  |

Supplementary Table 4. Step 3 of a forward-inclusion backward-elimination covariate selection. Backward elimination of CYP2C19*2 on FM.

| Pharmacokinetic parameter | Covariate | Drop in OFV | p | Significant? |
| --- | --- | --- | --- | --- |
| FM | CYP2C19*2 | -13.82742 | 0.0002 | YES |
